# The cost of promiscuity: sexual transmission of *Nosema* microsporidian parasites in polyandrous honey bees

**DOI:** 10.1038/srep10982

**Published:** 2015-06-30

**Authors:** K. E. Roberts, S. E. F. Evison, B. Baer, W. O. H. Hughes

**Affiliations:** 1 School of Biology, University of Leeds, Leeds, LS2 9JT, UK; 2 Centre for Integrative Bee Research, University of Western Australia, Crawley, WA 6009, Australia; 3 School of Life Sciences, University of Sussex, Brighton, BN1 9QG, UK

## Abstract

Multiple mating (and insemination) by females with different males, polyandry, is widespread across animals, due to material and/or genetic benefits for females. It reaches particularly high levels in some social insects, in which queens can produce significantly fitter colonies by being polyandrous. It is therefore a paradox that two thirds of eusocial hymenopteran insects appear to be exclusively monandrous, in spite of the fitness benefits that polyandry could provide. One possible cost of polyandry could be sexually transmitted parasites, but evidence for these in social insects is extremely limited. Here we show that two different species of *Nosema* microsporidian parasites can transmit sexually in the honey bee *Apis mellifera*. Honey bee males that are infected by the parasite have *Nosema* spores in their semen, and queens artificially inseminated with either *Nosema* spores or the semen of *Nosema*-infected males became infected by the parasite. The emergent and more virulent *N. ceranae* achieved much higher rates of infection following insemination than did *N. apis*. The results provide the first quantitative evidence of a sexually transmitted disease (STD) in social insects, indicating that STDs may represent a potential cost of polyandry in social insects.

Understanding the evolution of multiple mating (and insemination) by females with different males (polyandry) is a major theme in behavioural ecology. Genetic methods have revealed that polyandry is widespread across the animal kingdom^1^, and there is now strong empirical evidence that females can gain a variety of non-mutually exclusive direct material and indirect genetic benefits from polyandry^2^,^3^,^4^. The eusocial hymenopteran insects (ants, some bees and some wasps) are particularly interesting for understanding the evolution of polyandry because they show amongst the most extreme levels of polyandry of any animal (e.g. *Apis dorsata* honey bees can mate with as many as a hundred males in a couple of hours^5^), and have provided some of the best evidence for genetic benefits from polyandry. Social insect queens do not gain material benefits from polyandry, such as nuptial gifts or paternal care, nor do they appear to require additional matings to provide a sufficient sperm supply to fertilise their eggs^6^. However, there is now abundant theoretical and empirical evidence that polyandry can allow queens to produce genetically diverse offspring colonies that are fitter because they are more resistant to disease, have more optimum division of labour, and are less vulnerable to the impact of genetically incompatible matings^7^,^8^,^9^,^10^,^11^,^12^,^13^.

Although most attention has been directed at identifying benefits of polyandry, the accumulated abundance of evidence for such benefits arguably now means that the biggest question is not why females are polyandrous, but why females in so many species are not? In some cases monandry can be readily explained by a lack of available males, but in many more it cannot. In some cases monandry can be readily explained by kin selection when taxa are only facultatively eusocial or have reproductively totipotent females, but there are very many obligately eusocial taxa in which females are also monandrous^14^. Presumably the benefits of polyandry are outweighed in monandrous taxa by the costs of polyandry, such as energy expenditure, exposure to predators, direct harm by males, or sexually transmitted diseases. For example, the ejaculates of *Drosophila melanogaster* males include accessory compounds that reduce female survival, representing a direct cost of multiple mating in this species^15^. However, our knowledge of the strengths and relative importance of the costs of polyandry is still quite limited compared to our knowledge of the benefits. This is particularly the case for the social insects. Unlike most animals, the eusocial Hymenoptera are ancestrally monandrous, which is generally accepted to have been essential for the evolutions of eusociality in this group^14^,^16^,^17^. Polyandry has evolved as a derived state in approximately a third of social insects and reaches high levels (effective mating frequency of >2) in ten clades, but two thirds of the species investigated with sensitive genetic methods appear to be obligately monandrous^14^. Why this is the case is currently unclear. An evolutionary reversion to single mating in a socially parasitic leaf-cutting ant species, reduced immune function in polyandrous queens of another leaf-cutting ant species, and concave relationship between polyandry and fitness in a bumblebee suggest that polyandry can be costly to social insect queens^18^,^19^,^20^, but the nature of these costs are almost entirely unknown.

Theory predicts that the evolution of a polyandrous mating system should select for the coevolution of sexually transmitting parasites to exploit it^21^. The honey bee *Apis mellifera* is one of the most promiscuous of social insects, with queens mating with 12 males on average^22^, and two artificial insemination experiments with small numbers of queens (3 or 5) have suggested that deformed wing virus can transmit sexually in this species^23^,^24^. However, in spite of extensive investigation of host-parasite interactions in social insects^25^, no other sexually transmitting parasites are known in the group, and there has indeed been relatively limited study of such parasites in insects in general^21^. Here we investigate experimentally using artificial insemination and quantitative PCR whether two common, microsporidian parasites, *Nosema apis* and *N. ceranae*, can transmit sexually in honey bees. *N. apis* has coevolved with *A. mellifera* while *N. ceranae* is an emerging parasite following a host-jump from the Asian honey bee *A. ceranae*^26^. Both parasites can significantly reduce the fitness of honey bee colonies, but *N. ceranae* can be more virulent depending on host phenotype and age, has been associated with substantial colony losses in some (but not all) areas and appears to be spreading in honey bees^27^,^28^,^29^,^30^,^31^,^32^,^33^, and also spreading following a second host-jump in bumblebees^34^,^35^,^36^,^37^,^38^. Both *Nosema* species are faecal-orally transmitting parasites^26^, but whether they may transmit sexually as well is unknown.

## Results

### Sexual transmission: parasite presence in semen

**Figure 1:**
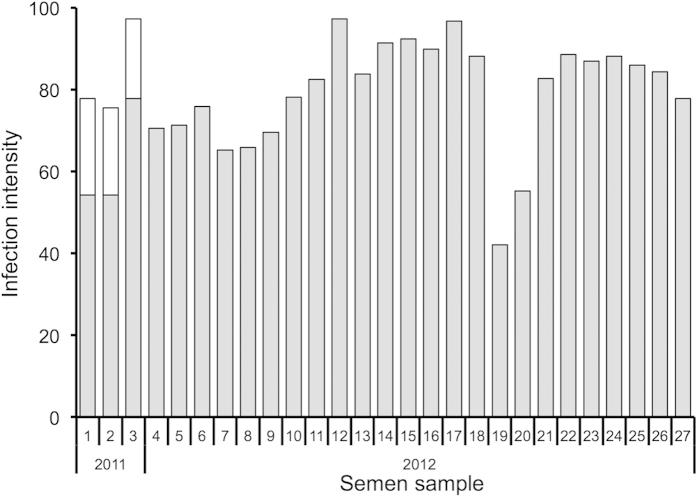
The intensity of *Nosema ceranae* (grey) and *N. apis* (white) infections in the semen of honey bee males. Data presented are for the 30/39 *Nosema*-positive samples of semen collected in 2011 and 2012, each of which was a pooled sample of semen from five males from a single colony, with all colonies having been previously confirmed to be infected with *Nosema*. Infection intensity is the number of spore-equivalents based on quantitative PCR (the number of parasite genes quantified in the sample transformed into the equivalent number of spores based on standard curves for a dilution series of extractions from known quantities of spores).

*Nosema* was present in 69% (27/39) of the semen samples examined, with 3/12 samples from 2011 and 24/27 samples from 2012 being positive. Semen sampled in 2011 had both *N. apis* and *N. ceranae*, but with much lower intensities of *N. apis*, while semen sampled in 2012 had only *N. ceranae* (Fig. 1). The level of *N. ceranae* infection did not differ significantly between colonies (*F*_22,1_ = 1.36, *P* = 0.256).

### Sexual transmission: insemination with parasite spores

**Figure 2:**
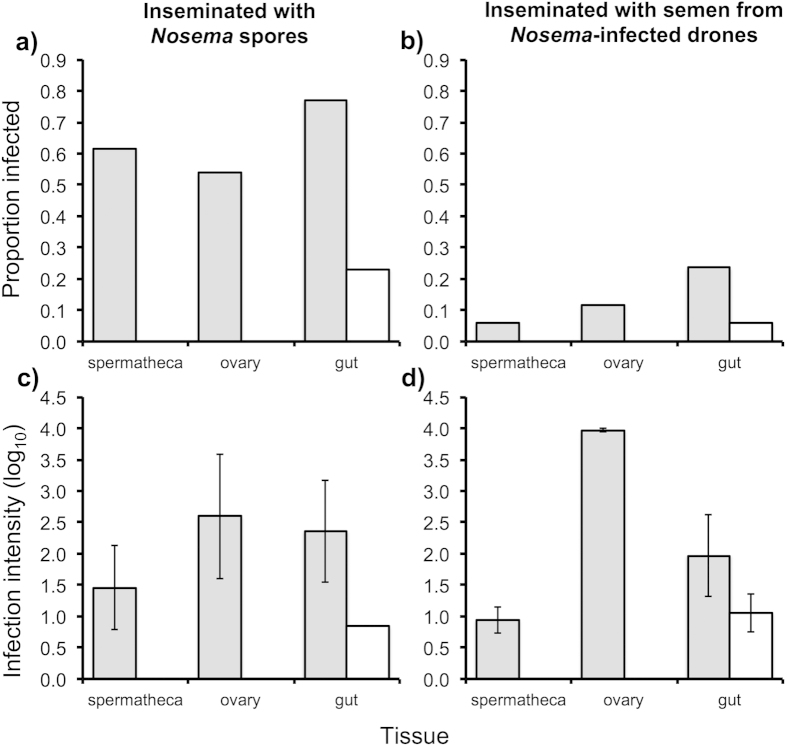
The prevalence (a,b) and mean ± s.e. intensity (**c**,**d**) of infections by the *Nosema ceranae* (grey) and *N. apis* (white) microsporidian parasites in spermathecae, ovaries and guts of honey bee queens that were artificially inseminated with either a mixed spore suspension of *Nosema apis* and *N. ceranae* (**a**,**c**) or semen from *Nosema*-infected males (**b**,**d**). Infection intensity is the log_10_ number of spore-equivalents, based on quantitative PCR (the number of parasite genes quantified in the sample transformed into the equivalent number of spores based on standard curves for a dilution series of extractions from known quantities of spores). The prevalence and intensities of infections differed significantly between *N. apis* and *N. ceranae* (*F*_44,1_ = 5.47, *P* = 0.024), and between tissues for *N. apis* (*F*_17,3_ = 151.7, *P* < 0.001), but not between tissues for *N. ceranae* (*F*_21,3_ = 2.48, *P* = 0.088).

No *Nosema* was detected in any of the tissue samples (spermatheca, ovary, gut) from any of the control queens that had been inseminated with sterile semen diluent. Ten of the 13 queens inseminated with *Nosema* spores were found to subsequently be positive for *N. apis* and/or *N. ceranae* (Fig. 2). The prevalence and intensities of infections differed significantly between *N. apis* and *N. ceranae* (*F*_44,1_ = 5.47, *P* = 0.024), and between tissues for *N. apis* (*F*_17,3_ = 151.7, *P* < 0.001), while the difference between tissues for *N. ceranae* was nonsignificant (*F*_21,3_ = 2.48, *P* = 0.088). Infections of *N. ceranae* were far more prevalent and intense than those of *N. apis* and were found in all tissues, whereas those of *N. apis* were found only in the gut (Fig. 2a,c). The intensities of the infections were highly variable, but the number of spore equivalents detected in the gut, ovary and spermathecal samples of three, two and one queen respectively, were more than an order of magnitude greater than the number inseminated (ca. 10,000 spores), indicating that the parasite had successfully established an infection and replicated.

### Sexual transmission: insemination with infected semen

No *Nosema* was detected in any of the tissue samples (spermatheca, ovary, gut) from any of the control queens. Queens inseminated with *Nosema*-infected semen were subsequently found to be positive for *Nosema* (Fig. 2), but at a much lower frequency than found in the previous experiment when the queens were inseminated with *Nosema* spores (Fig. 2b,d). For queens inseminated with *Nosema*-infected semen, *N. apis* was again detected only as low intensity infections in the guts of a small proportion of queens, while *N. ceranae* was detected in the spermatheca, ovary and guts of queens, with the highest prevalence and intensity of infections being in the guts. Infection intensities were again highly variable, with gut and ovary samples from three and two queens respectively having more than an order of magnitude more spores than were inseminated.

### Vertical transmission

None of the 400 eggs laid by queens that were either naturally infected with *Nosema*, or had been inseminated with semen containing *Nosema*, were found to carry the parasite.

## Discussion

The results show that *Nosema* microsporidian parasites can transmit sexually in honey bees and provide the first quantitative evidence of the sexual transmission of a parasite in social insects. Both *N. ceranae* and *N. apis* were present as spores in the semen of males, and queens artificially inseminated with either *Nosema* spores or the semen of *Nosema*-infected males became infected by the parasite. The quantities of *Nosema* DNA found in many of the queens was greater than that with which they were inseminated, showing that the parasite had replicated within the host. The results do not show whether infection was via the spermatheca or via ingestion of spores during post-insemination grooming, but they do show that insemination can result in infection. There was no evidence of subsequent vertical transmission of the parasites from queens to their eggs, but the presence of parasite infections in the guts would have made horizontal transmission possible.

The presence of *Nosema* spores in the semen of honey bee males is in keeping with the biology of other *Nosema* species, with large numbers of *N. bombi* spores having previously been found in the seminal vesicles of bumblebee males for example^39^,^40^, with the high infection rate in honey bee males^33^,^41^ and with a parallel study which found *N. apis* spores in the semen of honey bee males^42^. Our results demonstrate that queens can become infected by insemination with *Nosema* spores, but that the infection rate depends on species and probably dose. Only 23% and 6% of queens became infected with *N. apis* following insemination with either a *Nosema* spore suspension or semen from *Nosema*-infected males, respectively, compared to infection rates of 77% and 24% for *N. ceranae*. In addition, *N. apis* infections were limited to the gut of queens whereas *N. ceranae* infections spread to multiple tissues. It appears that *N. apis* may only be able to actively reproduce in the gut^43^, whereas *N. ceranae* is, like other *Nosema* species, able to infect a broader range of tissues^44^,^45^,^46^. Both the prevalence and intensity of infections following insemination with *Nosema* spore suspensions were greater than after insemination with semen from *Nosema*-infected males. The difference in infection intensities could be because infections were given less time to develop in the latter case, but the sensitivity of qPCR means this is unlikely to explain the difference in prevalence. The difference may simply be due to infections being dose-dependent, or could suggest that accessory gland compounds in semen reduce the viability of *Nosema* spores or upregulate the queen’s immune system.

The sexual transmission of a parasite can select for reduced virulence in order to enhance transmission^47^. In the case of honey bees, however, males are short-lived, can only mate once, and are provided with food and a protected environment by the workers in their natal colony, so selection on parasites for reduced virulence on male hosts is likely to be limited. In addition, if sexual transmission is dose-dependent, as suggested by the results here, then selection may act to maximise the number of spores in male ejaculates. Indeed, infection rates of *Nosema* in males appear to be particularly high and substantially reduces their fitness^42^,^33^. Selection should also act to reduce virulence in host females, particularly because queens are unusually long-lived in social insects^21^. *N. ceranae* has previously been found infecting both newly-eclosed and egg-laying queens, and can have important effects on their fitness^48^,^49^. However, queens are also provided with food and a protected nest environment by their workers, so selection here too may not be as strong as might initially seem the case.

The confirmation that *Nosema* parasites can transmit via insemination, regardless of whether it is via the spermatheca or post-insemination grooming, has important implications for our understanding of the evolution of polyandry. It means that infection by this parasite is a potential cost of multiple mating in honey bees, and quite probably also in bumblebees, given that *N. bombi* spores have previously been reported in the semen of bumblebee males and that *N. ceranae* is now a common parasite of bumblebees in some areas^35^,^36^,^37^,^38^,^39^. More generally, however, there are many other parasites that, like *Nosema*, use the faecal-oral route as their main mechanism of horizontal transmission (e.g. *Crithidia*, other protozoa, and some viruses), and the results suggest the possibility that some of them may also utilise sexual contact as a secondary mode of transmission. Clearly sexual transmission will not be the only, or even the primary, mode of transmission for parasite such as *Nosema*, and the risk of infection may not necessarily be the primary reason why so many species have not evolved monandry, but the results do suggest that sexually transmitted parasites can be one potential cost of polyandry. It would be worthwhile for future work to investigate the potential for sexual transmission by more parasites in order to gain a better understanding of the potential cost that sexually transmitted parasites may pose to females, and the significance of sexually transmitted parasites in explaining why so many social insects, and other animals, are essentially monandrous in spite of the significant benefits that polyandry can bring.

## Methods

Bees were obtained from managed colonies of *Apis mellifera carnica* honey bees, from an apiary in West Yorkshire. Colonies were checked for *Nosema* infection by examining the guts of 30 adult workers by microscopy and by Taqman qPCR, using primers specific to *N. apis* and *N. ceranae* to identify species (see below).

### Sexual transmission: parasite presence in semen

Sexually mature males were collected from 12 colonies in May-August 2011, and 27 colonies in May-June 2012, that had been confirmed by microscopy and quantitative PCR (qPCR) to be infected with *Nosema.* Semen was carefully harvested from drones using a Schley insemination apparatus, avoiding contamination with gut contents or any other tissues. The endophallus was fully everted by applying pressure to the drone thorax and semen released by squeezing the abdomen laterally from the head towards the abdomen, with the semen then being collected in pulled glass capillaries containing sterile phosphate buffered saline solution (PBS). Semen from five males per colony were pooled to give ca. 6 μl of semen per sample and placed into 90% ethanol at –20 °C for later analysis. A total of 39 pooled semen samples were screened over two years for parasite presence.

### Sexual transmission: insemination with parasite spores

A suspension of *Nosema* spores was obtained using bees from eight colonies that had been confirmed by microscopy and qPCR to be infected with both *N. apis* and *N. ceranae*. The guts of adult workers were homogenised in ddH_2_O, purified by Percoll centrifugation^50^, and the concentration of *Nosema* spores quantified using FastRead disposable haemocytometers (Immune Systems). The purified spore suspension was then made up to a dose of 1,667 spores per μl in sterile semen diluent (NaCl_2,_ C_6_H_12_O_6_, L +Arginine HCl, L-lysine, Tris Base, pH 8). Honey bee queens were reared from colonies in which no *Nosema* infections were detected. Young larvae (1-2 days old) were transferred into plastic queen cell cups (E.H. Thorne Beehives Ltd.) that were then placed in queenless, *Nosema*-free foster colonies where they were reared to adulthood. When cells were capped prior to pupation, queen cages were placed around the cells to protect them. Freshly eclosed queens were collected and kept in an incubator at 34 °C, 60% RH with escort workers that were uninfected with *Nosema* and 50% sucrose solution provided *ad libitum*, until they reached sexual maturity at seven days post-eclosion. Thirteen sexually mature queens were anaesthetized with CO_2_ and artificially inseminated with 6 μl of the *Nosema* spore suspension (ca. 10,000 spores), while five queens were inseminated with 6 μl of sterile semen diluent as controls. The queens were then placed in a cage with 20 two-day-old worker bees that were uninfected by *Nosema*, and kept in an incubator at 34 °C and 60% RH with an *ad libitum* supply of 50% sucrose solution. The survival of queens was monitored daily for 25 days. At the end of this period or on day of death, the gut, ovaries and spermatheca were dissected from queens and stored separately in 90% ethanol at –20 °C for later analysis.

### Sexual transmission: insemination with infected semen

Semen was harvested from sexually mature males that were collected from *Nosema*-infected colonies, with the infection status of males being subsequently confirmed in all cases by individual qPCR. Semen was harvested into capillaries from sets of ten males, giving ~6 μl of semen per capillary, with capillaries then sealed with sterile PBS and petroleum jelly, and stored at room temperature in a dark container until use. Seventeen sexually mature virgin queens were reared as described above and artificially inseminated with ~6 μl of drone semen using a Schley Insemination apparatus and a standardised method used widely for honey bee breeding, while 15 queens were inseminated with 6 μl of sterile PBS as controls. The queens were then housed with attendant workers as above for 14 days. Queens were dissected at the end of this period or on death, with gut, ovaries and spermatheca stored separately in 90% ethanol at –20 °C.

### Vertical transmission

Eggs were collected from five colonies of *A. mellifera* that had been confirmed by qPCR to be infected with *Nosema*, and from three colonies headed by queens artificially inseminated with semen from *Nosema*-infected males. Fifty eggs were collected from each colony and stored in 90% ethanol at –20 °C for later analysis.

### Molecular analysis

DNA was extracted by optimised methods for each tissue type. DNA extraction from semen was carried out by homogenising it in 0.5 ml of lysis buffer (100 mM Tris, pH 8.0, 10 mM EDTA pH 8.0 and 1% SDS, 2% Antifoam B emulsion) with 0.5 ml of 0.5 mm zirconia/silica beads (Thistle Scientific) using a Tissue Lyser LM (Qiagen) for 5 min at full speed. An aliquot of 75 μl of the homogenate was then boiled with 75 μl of 5% Chelex 100 (Biorad) suspended in 10 μM Tris Buffer. After centrifugation the supernatant was stored at −20 °C for molecular analyses. Queen tissues and eggs were extracted using a similar method, but were rinsed before homogenisation in autoclaved distilled water, washed twice in 5% bleach solution, and again rinsed in distilled water to remove any potential surface contamination of *Nosema*^51^. Tissues were homogenised in the Tissue Lyser for 3 min at full speed, a 75 μl aliquot of the homogenate was then incubated for 12 hours with proteinase K (Promega; 5 μl/ml) at 56 °C to aid spore wall degradation. Eggs were homogenised in groups of five per well, in 40 μl of 5% Chelex 100 (Biorad) solution suspended in 10 μM Tris Buffer whilst 75 μl of the tissue homogenates were boiled with 75 μl Chelex solution. After centrifugation the supernatant was stored at –20 °C for molecular analysis.

Genetic detection of *Nosema* was carried out using 1 μl of the DNA extracts with a StepOne Plus real-time PCR thermal cycler (ABI) with Taqman^®^ Universal Master Mix with UGA (ABI). We used the primers and probes for *N. apis* and *N. ceranae* designed by Bourgeois *et al*.^52^, and used the *A. mellifera β-actin* gene as an internal control^53^, after modification for use with Taqman^®^ by the design of a Molecular-Groove Binding Non-fluorescence Quencher (MGBNFQ) probe (NED-MGBNFQ- AAT TAA GAT CAT CGC GCC AC) using the Primer 3 program. *Nosema* infection was quantified using the standard curve method with known spore counts extracted to construct the standard curves. Six standard samples of both *N. apis* and *N. ceranae* were made in log dilutions of 1 × 10^6^ to 10 spores. The quantity of parasite gene number was normalised against a host control gene to control for variation in tissue quantity. Efficiencies for the target genes over four-fold dilutions were 96.8% for host, 96.9% for *N. apis* and 90.4% for *N. ceranae*. All samples were run in triplicate, with any repeats with high standard deviations (>0.5 C_t_) removed from the analysis. Plates were run with triplicate standard curve samples for each target assay to quantify parasite loads, and with triplicate negative controls to check for contamination.

### Statistical Analysis

All analysis was carried out in R 2.14.2. Intensities of *Nosema* infections, as determined by qPCR, were analysed using generalized linear models with a quasipoisson error structure to account for overdispersion, fitting infection intensity as the response variable and colony, parasite or tissue type as the independent variable.

## Additional Information

**How to cite this article**: Roberts, K. E. *et al*. The cost of promiscuity: sexual transmission of Nosema microsporidian parasites in polyandrous honey bees. *Sci. Rep.* **5**, 10982; doi: 10.1038/srep10982 (2015).
